# Complete Mitochondrial Genome Sequencing of *Brachypelma albiceps* and Comparative Codon Usage Bias Analysis Across Seven Mygalomorphae Species

**DOI:** 10.3390/biology15010016

**Published:** 2025-12-20

**Authors:** Qingbin Zhan, Yin Tang, Yang Zhao, Senlin Hou, Yalin Huang, Xiaoyan Zhao, Yunxia Chen, Xiaoming Xue

**Affiliations:** 1 Department of Criminal Science and Technology, Nanjing Police University, Nanjing 210023, China; zhanqb@njpu.edu.cn (Q.Z.); tangy@njpu.edu.cn (Y.T.); senlin_hou@163.com (S.H.); huangyl@njpu.edu.cn (Y.H.); 2 Key Laboratory of State Forestry and Grassland Administration on Wildlife Evidence Technology, Nanjing 210023, China; 3 Nanjing Institute of Agricultural Sciences in Jiangsu Hilly Area, Nanjing 210046, China; 20152601@jaas.ac.cn; 4 School of Grassland Science, Beijing Forestry University, Beijing 100083, China; zhao0223@bjfu.edu.cn

**Keywords:** codon usage bias, mitochondrial genome, *Brachypelma albiceps*, theraphosidae, phylogenomics, conservation genetics

## Abstract

Tarantulas (family Theraphosidae) are ecologically significant invertebrate predators in terrestrial ecosystems, but many species face threats from habitat fragmentation and unsustainable collection for the international pet trade. *Brachypelma albiceps*, a CITES Appendix II-listed species, lacks comprehensive mitochondrial genome characterization, limiting phylogenetic and evolutionary studies. Here, we report a complete mitochondrial genome sequence for *B. albiceps* (13,856 bp; GC content 32.84%) and provide detailed annotation. The genome exhibits typical metazoan mitochondrial organization, containing 13 protein-coding genes (PCGs), 22 tRNAs, and 2 rRNAs, with an AT-rich nucleotide composition (67.16%) characteristic of arthropod mitochondria. Comparative analyses of *B. albiceps* and six other Mygalomorphae species revealed strong biases toward A/T-ending codons and avoidance of G/C-ending codons. ENC–GC3s, neutrality, and PR2 analyses consistently indicate that natural selection plays a dominant role in shaping synonymous codon usage, with mutation pressure also contributing. Phylogenetic reconstruction based on 10 high-quality mitochondrial protein-coding genes from 23 spider species confirmed the placement of *B. albiceps* within the family Theraphosidae and its close phylogenetic relationship to *Cyriopagopus* species. These results provide valuable genomic resources for the Theraphosidae systematics, enhance our understanding of codon bias evolution, and provide critical DNA barcode data for forensic identification of CITES-regulated specimens in the illegal wildlife trade.

## Simple Summary

Tarantulas, including *Brachypelma albiceps*, are fascinating spiders known for their large body size, striking coloration, and docile temperament, making them popular in the international pet trade. However, habitat loss and over-collection threaten their wild populations. In this study, the complete mitochondrial genome of *Brachypelma albiceps* was sequenced to better understand its genetic composition and evolutionary history. The study specifically focused on analyzing codon usage patterns in the mitochondrial genome, providing insights into the evolutionary forces shaping mitochondrial gene expression. By comparing the mitochondrial genomes of various mygalomorph species, this study contributes to our understanding of their evolutionary relationships, genetic diversity, and the effects of natural selection and mutation pressure on their genomes. It provides essential molecular resources for species conservation and helps clarify the taxonomy of tarantulas, thereby advancing conservation efforts for these remarkable species.

## 1. Introduction

The family Theraphosidae Thorell, 1869, commonly known as tarantulas, is one of the largest and most diverse families of spiders (Araneae), comprising over 1000 described species in approximately 150 genera, distributed globally [1,2]. *Brachypelma* Simon, 1891, is one of the most iconic and commercially important genera in the tarantula family. Eugène Simon established this genus in 1891, with *Brachypelma emilia* (White, 1856) as the type species [3,4]. Many *Brachypelma* species are known for their vibrant coloration, particularly the distinctive red, orange, or yellow markings on the legs and carapace, and their exceptional lifespan. Because of these traits, they are among the most heavily traded tarantulas in the international pet trade [5,6]. Members of this genus are large, ground-dwelling terrestrial tarantulas characterized by their bright coloration, long lifespan, and docile behavior. Species such as *Brachypelma smithi* (F. O. Pickard-Cambridge, 1897), *B. hamorii* Tesmoingt, Cleton & Verdez, 1997, and *B. albiceps* are among the most well-known representatives and constitute the majority of *Brachypelma* specimens in the pet trade [7,8,9]. Because of their restricted distribution and popularity in the pet trade, several *Brachypelma* species are listed under Appendix II of the Convention on International Trade in Endangered Species (CITES) and are considered threatened due to habitat degradation and overcollection [7]. The phylogenetic position and taxonomic classification of *Brachypelma* have undergone substantial revision in recent years based on molecular evidence [10,11,12]. Critically, Turner et al. (2017) demonstrated through molecular phylogenetic analyses of mitochondrial DNA that both *Aphonopelma* Pocock, 1901, and *Brachypelma* are not monophyletic, with *Aphonopelma* recovered as polyphyletic and *Brachypelma* as paraphyletic [13]. Comprehensive phylogenetic analyses revealed that *Brachypelma* is not a monophyletic group but comprises two distinct clades, which were subsequently recognized as separate genera, *Brachypelma* and *Tliltocatl* Mendoza, 2020. The “red-legged” species remain in *Brachypelma* s.s., while the “red-rumped” species have been transferred to *Tliltocatl* [14,15]. This taxonomic revision resolved longstanding systematic uncertainties and provided a robust phylogenetic framework for evidence-based conservation prioritization within the CITES Appendix II-listed species. Both molecular and morphological studies have consistently placed *B*. *albiceps* within the family Theraphosidae within the infraorder Mygalomorphae, showing a close evolutionary relationship to other Central American *Brachypelma* species [15,16]. However, phylogenetic relationships within *Brachypelma* remain incompletely resolved due to limited molecular markers, with single-gene analyses often yielding low bootstrap support and conflicting topologies. The absence of complete mitochondrial genome sequences for most *Brachypelma* species further constrains phylogenomic approaches to resolving species relationships and evolutionary history within this conservation-priority genus.

The mitochondrial genome is widely used in studies of species classification, systematics, phylogeny, and evolutionary history because it is maternally inherited, evolves quickly, and has a conserved gene content and organization [17]. In spiders, research on the mitogenome has offered crucial insights into their evolutionary relationships, adaptive radiation, and biogeography [18,19]. Studies on the mitochondrial genomes of tarantulas are still limited, but their combination of high variability and conserved features makes them an excellent model system for studying spider evolution and taxonomy. For example, gene rearrangements in the mitogenome are quite common in spiders and may be linked to their unique evolutionary history [20]. Comparing mitochondrial genomes can also help clarify phylogenetic relationships within the tarantula family and provide molecular evidence for taxonomic revisions [21]. Furthermore, mitochondrial codon usage bias—the unequal use of synonymous codons—is common in invertebrates. For instance, insect mitochondrial genomes frequently exhibit a strong A + T bias [22].

To date, only three species of the family Theraphosidae have verified complete mitochondrial genomes deposited in GenBank: *Brachypelma albiceps*, *Cyriopagopus hainanus* (Liang, Peng, Huang & Chen, 1999), and *Cyriopagopus schmidti* (von Wirth, 1991) [23,24]. Furthermore, only seven species within the infraorder Mygalomorphae have verified complete mitochondrial genomes, including these three species of the family Theraphosidae. In this study, the complete mitochondrial genome of *B*. *albiceps* was sequenced and comprehensively annotated, representing the first complete mitochondrial genome of the genus *Brachypelma*. This research aims to (1) characterize the complete mitochondrial genome structure and composition of *B. albiceps*, (2) elucidate the codon usage patterns and their evolutionary significance of *B. albiceps* and its related taxa, (3) reconstruct high-resolution phylogenetic relationships using concatenated mitochondrial protein-coding genes, and (4) provide molecular resources to support conservation genetics and forensic identification for CITES-listed Theraphosidae species.

## 2. Materials and Methods

### 2.1. DNA Extraction and Genomic Sequencing

**Figure 1:**
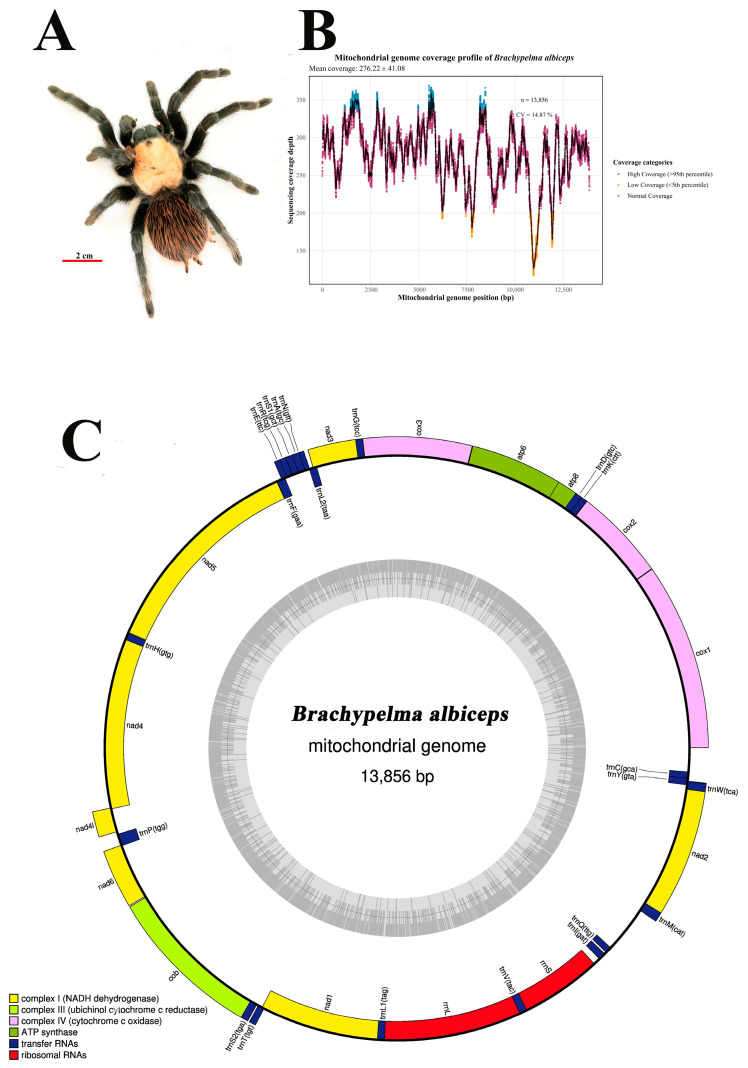
Morphological and genomic characterization of *Brachypelma albiceps*: (**A**) Adult specimen showing typical morphological features; (**B**) mitochondrial genome sequencing coverage profile displaying depth distribution across the 13,856 bp mitochondrial genome with mean coverage of 276.22 ± 41.08×; (**C**) circular representation of the complete mitochondrial genome organization. Genes transcribed clockwise are positioned within the inner circle, while those transcribed counterclockwise are located outside the circle. Gene categories are color-coded according to function: protein-coding genes (PCGs), transfer RNA genes (tRNAs), ribosomal RNA genes (rRNAs), and the control region.

The specimen of *Brachypelma albiceps* (Figure 1A) used in this study was obtained from the Tianjin Public Security Bureau on 8 June 2020, for species identification, as part of an investigation into a suspected violation concerning the endangerment of precious and protected wildlife. The specimen was confiscated from a private breeder located in Tianjin City (39.0851° N, 117.1994° E). The sample has been properly preserved in the Key Laboratory of Wildlife Evidence Technology, and its voucher number is NFPC2021-0930, ensuring the reliability and legality of the source of the sample. Total genomic DNA was extracted from the femoral muscle tissue of one leg of the specimen using the DNeasy Blood & Tissue Kit (Qiagen, Valencia, CA, USA). Tissue samples were preserved in 95% ethanol at −20 °C prior to extraction. DNA concentration and purity were assessed using a NanoDrop 2000 spectrophotometer (Thermo Fisher Scientific, Waltham, MA, USA), with A260/A280 ratios of 1.8–2.0 indicating high-quality DNA suitable for sequencing. Subsequently, a 150 base pair (bp) paired-end sequencing library was constructed, and high-throughput sequencing was performed using the Illumina NovaSeq 6000 platform (Illumina, San Diego, CA, USA) according to the manufacturer’s protocol. High-throughput Illumina sequencing generated 30,495,794 paired-end raw reads from the genomic DNA of *B. albiceps*. The coverage distribution was generally uniform, although a noticeable decline was observed around the 11,000 bp region (Figure 1B). The raw data were processed to remove adapters and low-quality bases using Trimmomatic version 0.39 [25]. After that, de novo assembly of the complete mitochondrial genome was conducted using the MITObim version 1.9.1 [26]. Genes were subsequently annotated using the MitoZ [27], and manual curation was performed with the assistance of Geneious version 2025.2 [28]. The complete mitochondrial genome sequence has been deposited in GenBank under accession number NC_062668 and is associated with BioProject PRJNA904502.

### 2.2. RSCU Calculation and Codon Usage Analysis

The mitochondrial genome sequences of seven mygalomorph species were retrieved from the National Center for Biotechnology Information (NCBI) GenBank database. Protein-coding genes were extracted from GenBank files using a custom Python script (Python v3.12.2).

Sequences were translated using the invertebrate mitochondrial genetic code, where ATA codes for methionine, TGA codes for tryptophan, and the AGA/AGG code for serine. The relative synonymous codon usage (RSCU) values were calculated using the standard formula: RSCU = (observed codon frequency)/(expected frequency under uniform usage), where expected frequency equals the total count of codons for an amino acid divided by the number of synonymous codons for that amino acid. Stop codons and single-codon amino acids (Met, Trp) were excluded from analysis. Codons were classified as strongly preferred (RSCU > 1.6), rarely used (RSCU < 0.6), or neutrally used (0.6 ≤ RSCU ≤ 1.6) based on commonly accepted thresholds in codon usage studies [29,30], which reflect significant deviation from uniform synonymous codon usage and indicate selection bias toward or against specific codons. Incomplete stop codons (single T or TA) observed in genes such as *COX2* and *ND4* were treated as complete stop codons following standard arthropod mitochondrial genome annotation conventions.

All analyses were conducted using R version 4.3.0 with the following packages: ggplot2 (visualization), pheatmap (heatmap generation), dplyr (data manipulation), FactoMineR (multivariate analysis), and corrplot (correlation analysis). Python was used for initial data processing and GenBank file parsing. Summary statistics (mean, standard deviation, coefficient of variation, minimum, and maximum) were calculated for each codon across all species. Heatmaps were generated using hierarchical clustering with Euclidean distance and complete linkage to visualize codon usage patterns. Statistical significance was assessed using Pearson correlation coefficients for continuous variables and chi-square tests for categorical comparisons.

### 2.3. Effective Number of Codons and GC3s Content Analysis

We performed ENC-plot analysis by plotting the GC content at the third codon position (GC3s) of each gene on the x-axis and the ENC value on the y-axis. The standard equation for ENC is described as follows [31]: If codon preference is influenced solely by mutations, the ENC values will cluster around the standard curve. When codon preference is strongly influenced by selection, the ENC values will deviate further below the curve. Additionally, the ENC ratio (ENC ratio) was calculated to further assess the factors influencing codon preference, with the formula ENC ratio = (ENC_observed − ENC_expected)/(61 − ENC_expected) [32].

### 2.4. Neutrality Analysis

This approach examines the relationship between GC content at the third codon position (GC3) and the average of the first and second codon positions (GC12) to distinguish between neutral evolution and selection-driven codon optimization. Neutrality plots were constructed by plotting GC12 against GC3 for all mitochondrial protein-coding genes within each species. Linear regression analysis was performed to quantify the relationship, with the regression slope serving as the primary neutrality index. Under strict neutrality, the slope approaches unity (slope = 1), indicating that mutational pressure affects synonymous and non-synonymous sites equally. Conversely, slopes significantly deviating from unity indicate the influence of natural selection: slopes < 1 suggest that selection constrains variation at non-synonymous sites relative to synonymous sites, while slopes > 1 may indicate positive selection or relaxed constraint [33]. Statistical significance of deviations from neutrality was assessed using 95% confidence intervals for regression slopes, with slopes whose confidence intervals excluded unity considered to represent significant departures from neutral expectations. Additionally, we calculated Pearson correlation coefficients (r) to quantify the strength of association between GC12 and GC3, as strong correlations (|r| > 0.7) typically indicate predominant mutational influence, while weak correlations (|r| < 0.3) suggest selection predominance.

### 2.5. PR2-Plot Analysis

PR2-plot analysis is used to assess whether the composition of the third codon position affects codon usage bias. In the PR2-plot, the center point (0.5, 0.5) of the plot represents A = T and G = C, indicating that codon usage bias is entirely influenced by mutation pressure [34]. Vectors radiating from the center point represent the direction and extent of codon preference for each gene. Previous studies have shown that the development of codon usage patterns is correlated with the base composition of the “silent” sites of codons. PR2-plot analysis is widely used to evaluate the bias between A/T and C/G at synonymous codon positions, thereby revealing the impact of mutation, selection, and other factors on codon usage bias (CUB) patterns. This analysis is particularly useful for protein-coding genes with fourfold degenerate synonymous codons. Therefore, a scatter plot was constructed, with the y-axis representing A3s/(A3s + T3s) and the x-axis representing G3s/(G3s + C3s).

### 2.6. Phylogenetic Analysis

To reconstruct the phylogenetic relationships, we obtained mitochondrial genomes of 23 spider species representing the Mesothelae, Mygalomorphae, and Araneomorphae lineages, using two horseshoe crab species as outgroups (Supplementary Table S1). All 13 protein-coding genes (PCGs) were extracted per species, concatenated in standard order, and translated into amino acid sequences employing the invertebrate mitochondrial genetic code. Following a rigorous quality assessment based on gap ratio and conservation score, we excluded three poorly aligned genes (*ATP8*, *ND4L*, and *ND6*) due to unreliable homology inference. The ten remaining high-quality PCGs were individually aligned using MAFFT v7.505 with the L-INS-i algorithm [35], trimmed with trimAl v1.5 using strict parameters [36], and concatenated into a final dataset of 2456 amino acid positions (containing 1872 parsimony-informative sites; Supplementary File S2). We then performed maximum likelihood phylogenetic inference with IQ-TREE v2.3.6 [37], which implemented an automated partition scheme and model selection using ModelFinder under the Bayesian Information Criterion (BIC). ModelFinder evaluated candidate protein evolution models and assigned the best-fit substitution model to each gene partition. Branch support was evaluated using 1000 UFBoot2 replicates and SH-aLRT tests, with nodes achieving UFBoot ≥ 95% and SH-aLRT ≥ 90% considered strongly supported. The resulting tree was visualized using EvolView v2 [38]. The data files associated with the phylogenetic tree construction are provided in Supplementary Files S1–S4.

## 3. Results

### 3.1. Complete Mitochondrial Genomes of Brachypelma albiceps

The complete mitochondrial genome of *B. albiceps* was successfully assembled as a circular, double-stranded DNA molecule of 13,856 bp in length. The mitochondrial genome contains the standard metazoan gene complement of 37 genes: 13 protein-coding genes (PCGs), 22 transfer RNA genes, and 2 ribosomal RNA genes, along with a putative control region (Figure 1C). The 13 protein-coding genes include the standard complement found in arthropod mitochondria: *cox1-3*, *nad1-6* and *nad4L*, *atp6* and *atp8*, and *cytb*. The nucleotide composition showed the characteristic A + T bias typical of arthropod mitochondrial genomes, with the overall A + T content ranging from approximately 61.9% to 68.5% across different gene categories. The 22 tRNA genes displayed the expected diversity in size and secondary structure, with some showing deviations from the canonical cloverleaf structure commonly observed in spider mitochondrial genomes. The two ribosomal RNA genes (12S and 16S rRNA) maintained the conserved secondary structure elements essential for ribosome function, despite showing elevated A + T content compared to the protein-coding genes.

### 3.2. RSCU Analysis and Synonymous Codon Usage Patterns

**Figure 2:**
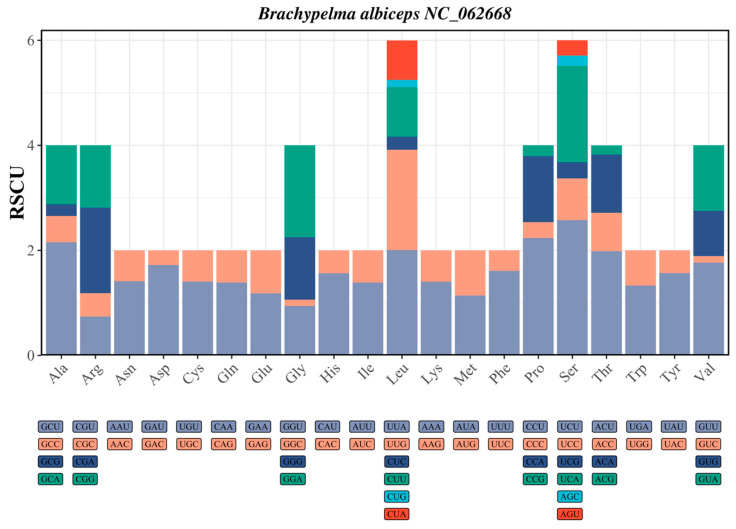
Relative synonymous codon usage (RSCU) analysis of the mitochondrial genome in *Brachypelma albiceps*.

The relative synonymous codon usage (RSCU) analysis of seven mygalomorph mitochondrial genomes revealed pronounced and non-random codon usage patterns characteristic of arachnid mitochondrial genomes, with RSCU values ranging dramatically from 0.047 (GUC, Val) to 3.536 (UUA, Leu) (Figure 2, Supplementary Table S2A–C), indicating strong codon bias that substantially exceeds the range typically observed in nuclear genomes and reflects the characteristic AT-rich composition of arthropod mitochondrial genomes. Eleven codons demonstrated strong usage preference (RSCU > 1.6), with the three most highly preferred being UUA, UCU, and CCU, suggesting strong evolutionary selection for translational efficiency, while twenty-two codons exhibited significantly reduced usage (RSCU < 0.6), with the most avoided being CUG, AGC, and GUC, particularly highlighting the extreme avoidance of CUG (CTG in DNA) across all mygalomorph species (average RSCU ≈ 0.10) as one of the most striking examples of codon avoidance in arthropod mitochondrial genomes. 

### 3.3. ENC-GC3s Analysis of Mygalomorphae Species Mitogenome

**Figure 3:**
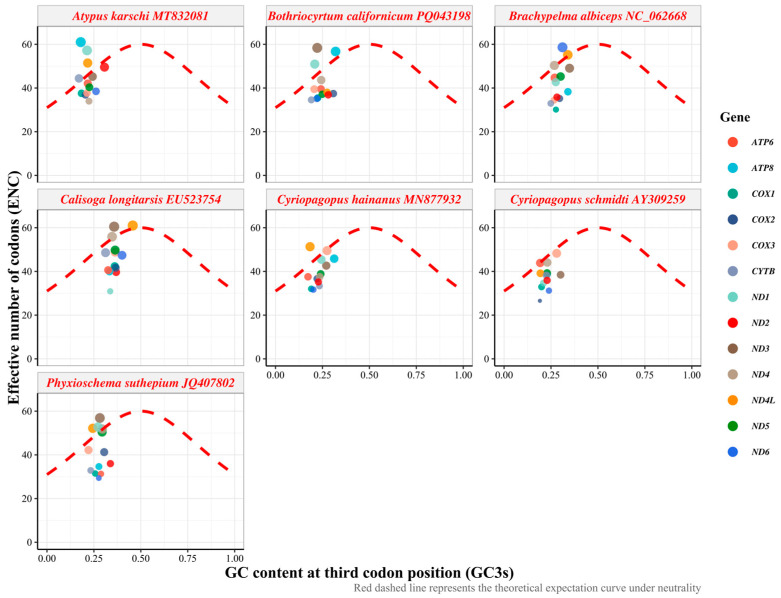
ENC-GC3s plot analysis of 13 PCGs from seven Mygalomorphae species. The red dashed line represents the expected curve when codon usage bias is influenced solely by mutation pressure under neutral evolution (ENC_expected = 2 + GC3s + 29/[GC3s^2^ + (1 − GC3s)^2^]).

Analysis of effective number of codons (ENC) and GC content at third codon positions (GC3s) was performed across 91 gene sequences (13 protein-coding genes across 7 taxa) from seven Mygalomorphae species to evaluate the relative contributions of mutational pressure and natural selection in shaping codon usage bias (Figure 3, Supplementary Table S3A–C). ENC values ranged from 26.56 to 61.00 across all genes and species, with an overall mean of 42.10 ± 8.34 and a median of 39.67. The relatively broad distribution of ENC values indicated substantial variation in codon usage bias intensity among mitochondrial genes. The ENC distribution exhibited positive skewness (0.57) and platykurtic characteristics (kurtosis = −0.51), suggesting predominance of moderately biased codon usage patterns with occasional instances of extreme bias or neutrality. GC3s values ranged from 0.170 to 0.457, with a mean of 0.265 ± 0.058 and a median of 0.257. The distribution showed positive skewness (0.66) and slight positive kurtosis (0.17), indicating concentration of values around the low-GC3s region characteristic of arthropod mitochondrial genomes. The mean GC3s content of 26.5% confirmed the pronounced A + T bias at synonymous third codon positions typical of invertebrate mitochondrial genomes. ENC-GC3s plot analysis revealed systematic deviations from the theoretical neutrality curve (Figure 3). Species-specific clustering patterns were evident, with *B*. *albiceps* genes distributed across ENC values of 28.95–55.27 and GC3s values of 0.193–0.345, while *A*. *karschi* exhibited a broader range (ENC: 26.56–61.00; GC3s: 0.170–0.307).

### 3.4. Mutational Pressure Constrained by Natural Selection

**Figure 4:**
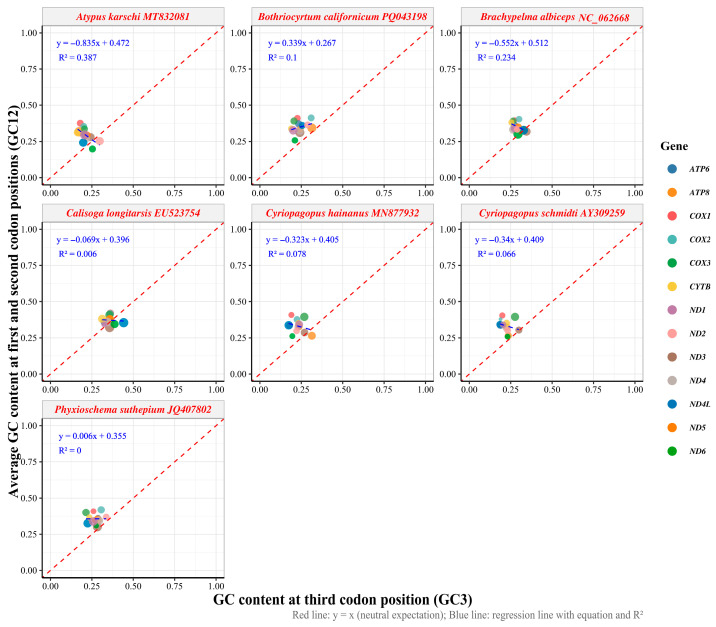
The correlation between GC content at the first and second codon positions (GC12) and GC content at the third codon position (GC3) across mitochondrial genomes of seven Mygalomorphae species.

Neutrality plot analysis was conducted to assess the relative contributions of mutation pressure and natural selection in shaping codon usage bias by examining the relationship between GC content at the first and second codon positions (GC12) and the third codon position (GC3) (Figure 4, Supplementary Table S4A–C). Under neutral evolution, a strong positive correlation between GC12 and GC3 indicates that mutation pressure dominates codon usage patterns, while a weak or absent correlation suggests the predominant influence of natural selection. The analysis revealed substantial interspecific variation in GC content and neutrality patterns across the seven mygalomorph species examined. GC3 content exhibited the greatest variability, ranging from 17.31% in *A. karschi* to 44.19% in *C. longitarsis*, while GC12 content showed more constrained variation from 19.72% to 41.89%. For *Brachypelma albiceps* specifically, the GC3 content ranged from 26.44% to 34.51% (with a mean of 29.14%), while the GC12 content ranged from 30.07% to 40.36% (with a mean of 35.12%).

Linear regression analysis of GC12 against GC3 yielded regression coefficients ranging from −0.84 to 0.34 across species, with R^2^ values ranging from 0 to 0.3873. The majority of species demonstrated weak correlations between GC12 and GC3, with only *A. karschi* showing a statistically significant relationship (slope = −0.84, R^2^ = 0.39, *p* = 0.023). *B. albiceps* exhibited a negative regression slope (−0.55) with moderate explanatory power (R^2^ = 0.23, *p* = 0.094), while other species, including *C. longitarsis* and *P. suthepium*, displayed slopes approaching zero with negligible R^2^ values.

### 3.5. Systematic Deviation from PR2 Parity Rules

**Figure 5:**
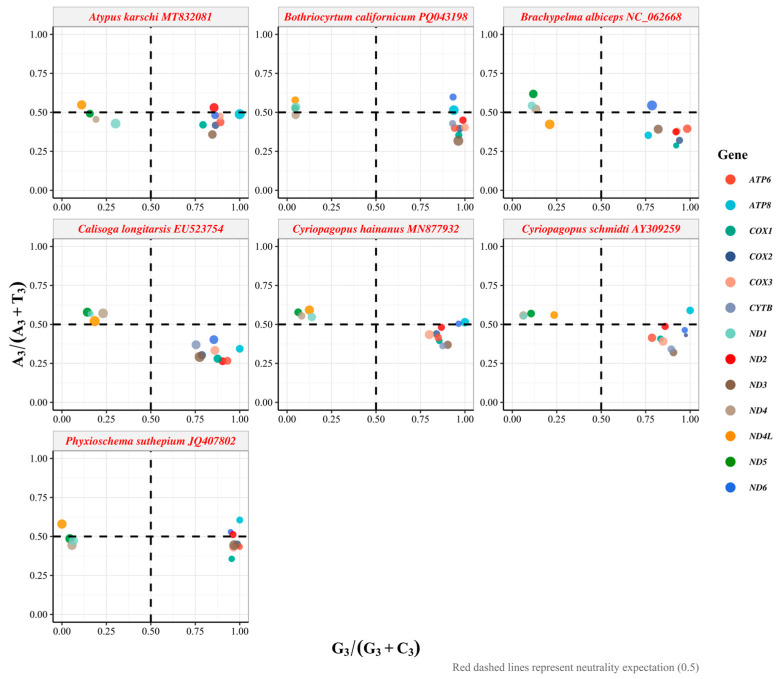
PR2-plot analysis of 13 PCGs in the mitochondrial genomes of seven Mygalomorphae species.

PR2-plot bias analysis examined compositional asymmetry at synonymous sites by plotting G_3_/(G_3_ + C_3_) against A_3_/(A_3_ + T_3_), where the center point (0.5, 0.5) represents neutrality under equal mutation rates (Figure 5, Supplementary Table S5A–C). Analysis of the seven mygalomorph species revealed pronounced heterogeneity in PR2 bias patterns across both species and genes. Species-specific mean G_3_/(G_3_ + C_3_) ratios ranged from 0.636 in *Brachypelma albiceps* to 0.678 in *Bothriocyrtum californicum*, while mean A_3_/(A_3_ + T_3_) ratios varied from 0.392 in *Calisoga longitarsis* to 0.478 in *Phyxioschema suthepium*. Within *B. albiceps*, individual genes exhibited substantial variation, with G_3_/(G_3_ + C_3_) ratios spanning 0.1098 to 0.9836 and A_3_/(A_3_ + T_3_) ratios ranging from 0.2876 to 0.6182. The predominant clustering of genes in the lower-right quadrant indicates a systematic preference for G over C and T over A at third codon positions. This asymmetry is particularly pronounced in *Atypus karschi*, with smaller deviations observed in other Mygalomorphae species.

### 3.6. Phylogenetic Relationship

**Figure 6:**
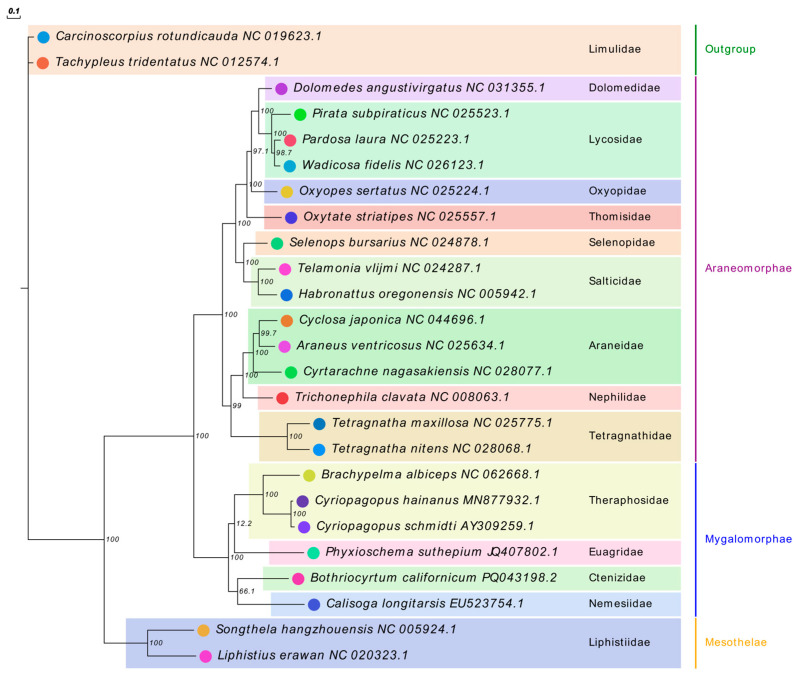
Phylogenetic tree of 23 spider species based on maximum likelihood analysis of concatenated mitochondrial protein-coding genes. Bootstrap support values (UFBoot/SH-aLRT) are indicated at nodes. The tree was rooted with two horseshoe crab outgroups (Xiphosura). Major lineages (Mesothelae, Mygalomorphae, and Araneomorphae) are indicated by colored branches.

Maximum likelihood phylogenetic analysis of concatenated mitochondrial protein-coding genes resolved the evolutionary relationships among 23 spider species, representing three major lineages (Mesothelae, Mygalomorphae, and Araneomorphae) with high bootstrap support (Figure 6). The resulting phylogenetic tree clearly delineates the evolutionary relationships between *Brachypelma albiceps* and other spider species, confirming its taxonomic placement within the Theraphosidae family. *B. albiceps* was unequivocally positioned within Theraphosidae, forming a robustly supported monophyletic clade with *Cyriopagopus hainanus* and *Cyriopagopus schmidti*. The phylogenetic placement of Theraphosidae within Mygalomorphae revealed it to be sister to a clade comprising Euagridae (*Phyxioschema suthepium*), Ctenizidae (*Bothriocyrtum californicum*), and Nemesiidae (*Calisoga longitarsis*), although internal relationships within Mygalomorphae showed varying levels of statistical support. The Araneomorphae lineage was represented by a well-supported monophyletic radiation comprising 15 species from 9 families with consistently high support values. Interfamilial relationships within Araneomorphae were strongly supported, with Lycosidae forming a well-supported clade, and Tetragnathidae occupying a derived position within the araneomorph tree.

## 4. Discussion

### 4.1. Structural and Compositional Deviations of the Mitochondrial Genome in *Brachypelma albiceps*

The complete mitochondrial genome of *Brachypelma albiceps* (13,856 bp, GC content 32.84%) exhibits the typical organization found in arthropod mitochondrial genomes, containing 13 protein-coding genes, 22 transfer RNA genes, and 2 ribosomal RNA genes, consistent with previous studies on spider mitochondrial genomes [21]. The genome exhibits a pronounced AT-rich composition, with an AT content of approximately 67.16%. This AT bias is consistent with the high AT content observed in most arthropod mitochondrial genomes, where the AT content typically ranges from 60% to 80% [21,39]. This AT bias reflects the combined influence of mutational pressure, natural selection, and optimization of translation efficiency during genome evolution [40,41]. The AT-rich phenomenon is especially pronounced in the protein-coding genes of *B. albiceps*, particularly at the start codon positions. Notably, we observed the coexistence of the typical ATG start codon alongside atypical start codons such as ATT, ATA, and TTG, highlighting the flexibility and diversity of the invertebrate mitochondrial translation system [42].

### 4.2. Synonymous Codon Preference in Seven Mygalomorphae Species

The analysis of relative synonymous codon usage (RSCU) revealed a pronounced bias in favor of codons ending in A/T, with notable preferences for arginine (AGA) and serine (UCU), both of which have high RSCU values. This preference for A/T-ending codons is typical in mitochondrial genomes, which are subject to evolutionary pressures such as mutation bias and selective optimization of translation efficiency [43]. The ENC analysis revealed that the ENC values of most protein-coding genes are significantly lower than the expected values under the theoretical neutral mutation-drift equilibrium, with ENC values clustering notably below the theoretical neutral curve [31,44]. This suggests that selection has surpassed the influence of mutation pressure, highlighting the predominant role of selection on synonymous codon usage. The neutrality plot analysis showed a weak or negative correlation between the GC content of synonymous sites (GC3) and non-synonymous sites (GC12) across the seven Mygalomorphae species examined, with R^2^ values ranging from 0 to 0.39. This indicated that natural selection has played a role in decoupling the compositional variation between synonymous and non-synonymous sites [33,34]. The PR2-plot analysis further indicates a significant deviation from PR2 at the third codon position in synonymous codons, with a bias towards G over C and T over A. This reflects the strand-specific mutations during mitochondrial genome replication and the selection for optimal tRNA–codon interactions [45,46]. A similar PR2-bias pattern has been reported in other arthropod mitochondrial genomes, for example, in hoverflies (Rhingia), where unequal A/T and G/C usage at third codon positions indicates the joint effects of mutation pressure and natural selection [47].

### 4.3. Phylogenomic Implications and Conservation Significance

The phylogenetic analysis recovered Mesothelae (*Songthela hangzhouensis* and *Liphistius erawan*) as the basal lineage, sister to a clade containing Mygalomorphae and Araneomorphae, consistent with established spider phylogeny [1,48]. Within Araneomorphae, the phylogenetic reconstruction revealed a well-supported monophyletic radiation comprising 15 species from 9 families, with strongly resolved interfamilial relationships. Molecular phylogenetic distance analysis further indicated considerable heterogeneity in evolutionary rates across spider lineages, as reflected by the total tree length and patterns of sequence divergence [47,48]. *Brachypelma albiceps* was placed within Mygalomorphae alongside *Phyxioschema suthepium*, *Cyriopagopus schmidti*, and *C. hainanus*, forming a well-supported monophyletic clade clearly differentiated from Araneomorphae. This placement is consistent with the morphological and molecular evidence supporting the monophyly of Theraphosidae [1,13,15]. However, it should be noted that this analysis confirms only the family-level placement of *B. albiceps* within Theraphosidae, rather than resolving intra-generic relationships, which would require denser taxon sampling within *Brachypelma* sensu stricto.

From a conservation perspective, the genomic data generated in this study provide essential baseline molecular resources for the management and protection of *B. albiceps* and related CITES-listed species [6,7]. The genus *Brachypelma* has experienced severe population declines due to habitat destruction and unsustainable collection for the international pet trade, resulting in the CITES Appendix II listing of all species [14]. The mitochondrial genome data generated here have several practical conservation applications. First, complete mitogenome sequences enable genetic diversity within and among populations, identification of evolutionarily significant units for prioritized conservation, and forensic identification of illegally traded specimens [7]. The codon usage bias patterns identified herein may also have implications for understanding adaptive evolution in response to environmental stressors, as synonymous codon usage has been shown to influence gene expression efficiency, protein folding kinetics, and stress tolerance in other invertebrate systems [43]. Second, species-diagnostic regions identified through comparative analysis can support forensic identification of illegally traded specimens, complementing COI-based DNA barcoding approaches [6,14]. Third, codon usage patterns may inform understanding of adaptive evolution, as synonymous codon usage has been shown to influence gene expression efficiency and stress tolerance in other invertebrate systems [43]. Future studies incorporating multiple georeferenced specimens from known wild populations across the species’ range in Mexico would strengthen the conservation utility of these data by enabling population-level genetic analyses and assessment of evolutionarily significant units for prioritized conservation [13,14].

## 5. Conclusions

In this study, we characterize the complete mitochondrial genome of *Brachypelma albiceps* and provide a comparative analysis of codon usage across seven Mygalomorphae species. Our findings demonstrate that natural selection primarily shapes synonymous codon usage patterns in mygalomorph mitochondrial genomes, with a strong preference for A/T-ending codons, reflecting optimization for translational efficiency under AT-rich constraints. Phylogenetic analysis confirms *B. albiceps*’ placement within the family Theraphosidae in the infraorder Mygalomorphae and supports the monophyly of major spider infraorders. Differential amino acid identity patterns across protein-coding genes’ functionally constrained regions, which can serve as reliable markers for species identification and population studies. The mitochondrial genome data generated in this study provide valuable molecular resources for the following: (1) Species identification: The complete mitogenome sequence enables the development of diagnostic markers for forensic identification of CITES-regulated specimens in illegal wildlife trade investigations. (2) Conservation genetics: These data establish a molecular baseline for future population genetic studies of *B. albiceps*, which will be essential for assessing genetic diversity and identifying evolutionarily significant units within this threatened species. (3) Evolutionary studies: The codon usage bias patterns characterized herein contribute to our understanding of the evolutionary forces shaping mitochondrial genome evolution in tarantulas.

However, limitations of this study should be noted. The analysis is based on a single captive-bred specimen of unknown geographic provenance within Mexico. Future studies incorporating multiple georeferenced specimens from wild populations across the species’ native range would strengthen the conservation utility of these genomic resources and enable more robust inferences about population structure and evolutionary history.

## Data Availability

The mitochondrial genome of *Brachypelma albiceps* has been deposited at NCBI BioProject under the accession PRJNA904502 (https://www.ncbi.nlm.nih.gov/bioproject/904502, accessed on 12 September 2025). The data that support the findings of this study are available in the National Center for Biotechnology Information (NCBI) at GenBank with accession number NC_062668 (https://www.ncbi.nlm.nih.gov/nuccore/NC_062668.1/, accessed on 12 September 2025).

## Supplementary Materials

The following supporting information can be downloaded at: https://www.mdpi.com/article/10.3390/biology15010016/s1. Table S1. Spider species and corresponding mitochondrial genome accession numbers used in this study. Table S2: A: Summary statistics of relative synonymous codon usage (RSCU) for mitochondrial protein-coding genes in seven mygalomorph species. B: Amino acid-specific summary statistics of relative synonymous codon usage (RSCU). C: Complete dataset of relative synonymous codon usage (RSCU) for individual codons. Table S3: A: Summary statistics of effective number of codons (ENC) and GC content at third codon position (GC3s) for mitochondrial protein-coding genes across seven mygalomorph species. B: Gene-specific summary statistics of effective number of codons (ENC) and GC content at third codon position (GC3s) for mitochondrial protein-coding genes. C: Complete dataset of ENC and GC3s values for all mitochondrial protein-coding genes in seven mygalomorph species. Table S4: A: Summary statistics of GC content at different codon positions and effective number of codons (ENC) for mitochondrial protein-coding genes in mygalomorph species. B: Linear regression analysis of GC12 against GC3 for mitochondrial protein-coding genes in mygalomorph species. C: GC content at different codon positions and effective number of codons (ENC) for individual mitochondrial protein-coding genes in mygalomorph species. Table S5: A: Summary statistics of purine–pyrimidine ratio 2 (PR2) bias and effective number of codons (ENC) for mitochondrial protein-coding genes in mygalomorph species. B: Gene-specific summary statistics of purine–pyrimidine ratio 2 (PR2) bias and effective number of codons (ENC) for mitochondrial protein-coding genes across mygalomorph species. C: Complete dataset of purine–pyrimidine ratio 2 (PR2) bias and effective number of codons (ENC) for individual mitochondrial protein-coding genes in mygalomorph species. Supplementary Files S1–S4: S1: Raw_Sequences: The original nucleotide/protein sequences downloaded from GenBank. S2: The results of multiple sequence alignment performed by MAFFT. S3: Trimmed final multiple sequence alignment. S4: All output files from the IQ-TREE run, including the maximum likelihood tree (*.treefile) and the log file.
